# Iatrogenic Pseudoaneurysm of the Lateral Femoral Circumflex Artery Following Antegrade Intramedullary Femur Nailing: A Case Report

**DOI:** 10.7759/cureus.52009

**Published:** 2024-01-10

**Authors:** Nur Ayuni Khirul Ashar, Imma Isniza Ismail, Rahul Lingam

**Affiliations:** 1 Orthopedic and Traumatology Department, Universiti Teknologi MARA, Kuala Lumpur, MYS; 2 Orthopedic Department, Universiti Putra Malaysia, Kuala Lumpur, MYS; 3 Orthopedic Department, Hospital Serdang, Kuala Lumpur, MYS

**Keywords:** trauma, vascular injury, lateral femoral circumflex artery, intramedullary femur nailing, iatrogenic pseudoaneurysm

## Abstract

Few cases of femoral artery pseudoaneurysm or its branches have been reported following intramedullary nailing of femur fractures. The occurrence of a false aneurysm of the lateral femoral circumflex artery (LFCA) for such a fracture has never been reported so far. We report a case of a young male with a delayed presentation of right thigh swelling following an antegrade interlocking nail femur. Ultrasonography and CT angiography of the right thigh confirmed a pseudoaneurysm communicating with LFCA. After unsuccessful ultrasound-guided compression therapy, the patient underwent embolization of the right LFCA pseudoaneurysm by an interventional radiologist. Although intramedullary nailing is considered a safe procedure, a pseudoaneurysm should be suspected when the patient presents with a painful swelling after the surgery. Glue embolization of the artery should be considered as one of the treatment options, as it yields favorable outcomes with less morbidity.

## Introduction

This article was previously presented as a meeting abstract at the 50th Malaysian Orthopaedic Association Annual Scientific Meeting on June 22, 2021.

Pseudoaneurysm, also known as false aneurysm, is formed by the initial collection of blood as a result of a damaged arterial wall. This is followed by the formation of a hematoma, its containment by the surrounding tissues, and the inflammatory process [1]. Few cases of femoral artery pseudoaneurysm or its branches have been reported following closed reduction and internal fixation of both proximal [2-4] and distal femur fractures [5]. Traumatic false aneurysm following open reduction for femoral shaft fracture has also been reported previously [6]. However, none have documented the incidence of a pseudoaneurysm of the lateral femoral circumflex artery (LFCA) following closed reduction and intramedullary nailing for a midshaft femur fracture.

## Case presentation

**Figure 1:**
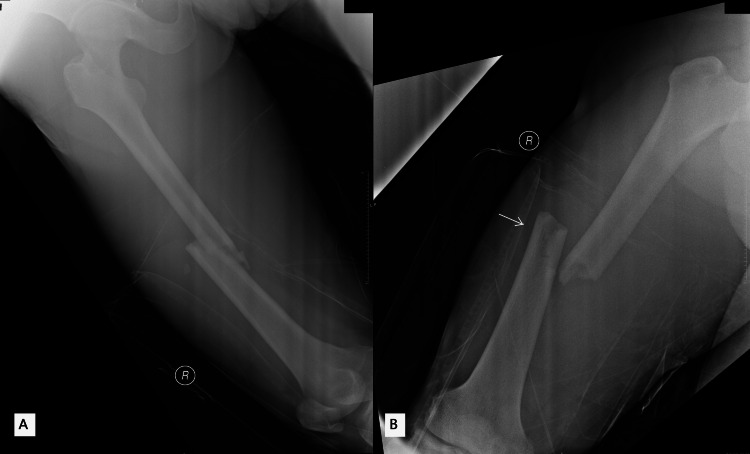
Plain radiographs of the right femur in lateral and anteroposterior (AP) views (A and B) reveal a midshaft right femur fracture with anterolateral displacement of the distal part, as indicated by the white arrow.

A 26-year-old Burmese male was involved in a motor vehicle accident between his motorcycle and a car. He was brought to the emergency department by ambulance with a complaint of pain and deformity in his right lower limb. Upon assessment, he was diagnosed with a closed fracture of the midshaft right femur (Figure 1) and an ipsilateral distal tibia fracture. Additionally, he sustained a deep laceration wound over the ipsilateral knee. As a part of immediate management, he underwent an emergent debridement and suturing of the wound, along with the insertion of a calcaneal pin. While in the ward, he was placed on 5 kg of skeletal traction.

Definitive surgery was delayed due to the prolonged waiting time for the operation. Ten days later, the patient underwent internal fixation for both femoral and tibia fractures. Before the surgery, there were no signs of infection in the wound over the right knee, and the compartments of the right thigh and leg were soft, with no signs of vascular compromise. In the operating room, the patient was placed in a supine position on a radiolucent traction table, and the right leg was set on a below-knee back slab before applying the traction boot.

**Figure 2:**
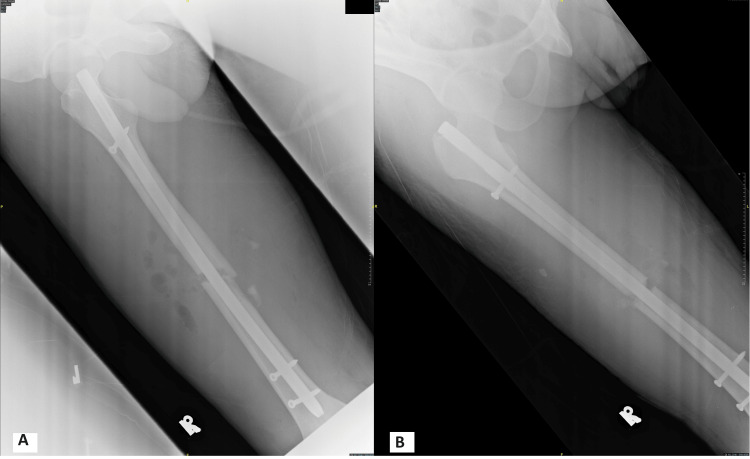
Post-operative plain radiographs of the right femur in oblique and anteroposterior (AP) views (A and B) show the interlocking nail femur in situ with a satisfactory fracture reduction.

The antegrade nailing of the right femur was performed first. A threaded guide wire was carefully inserted over the tip of the greater trochanter to establish the entry point, followed by the use of an awl to open the medullary canal. Subsequently, a ball-tip guide wire was advanced until it reached just proximal to the fracture site. The fracture was then reduced through a combination of manual manipulation by the assistant and tractional force applied by the traction table. The surgeon further introduced the guide wire, followed by sequential canal reaming. An 11-mm nail was then inserted, and the procedure was completed by securing proximal and distal screws (Figure 2). The patient was subsequently placed in a supine position, and a minimally invasive plate osteosynthesis was performed using an anterolateral locking plate to address the extraarticular fracture of the ipsilateral distal tibia.

The patient was discharged three days post-operatively, and no complications were encountered during or immediately after the procedure. However, one week following discharge, he presented to the casualty with a three-day history of pain and swelling over his right thigh. He denied any recent trauma to the thigh and had been compliant with non-weight-bearing crutches. At home, he remained afebrile, and there were no signs of wound dehiscence or discharge from the surgical wound.

On examination, there was localized swelling over the proximal anterior right thigh measuring 10 cm x 15 cm and located 10 cm distal to the inguinal crease. The swelling was firm, non-tender, and non-pulsatile, with no signs of infection. The surgical wounds were intact and clean. Notably, the swelling was situated lateral to the pulsation of the femoral artery. Pulsation of the posterior tibial artery was strong, while the dorsalis pedis artery had weaker pulsation. The patient’s toes appeared pink with an immediate capillary refill time. A plain radiograph revealed no evidence of implant loosening, implant breakage, or fracture displacement. His hemoglobin level was 10.7 g/dL, his white blood cell count was 8.9 x 10^9^/L, and his platelet level was within the normal range of 431 x 10^9^/L. The coagulation profile was also within the normal range.

**Figure 3:**
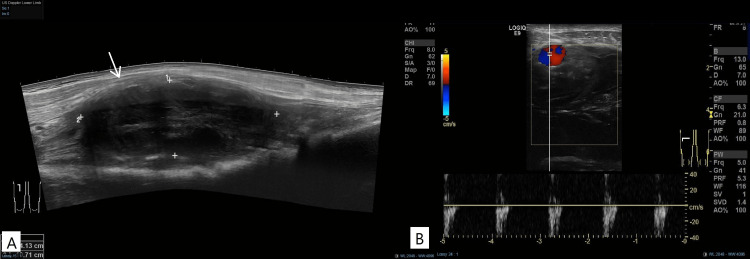
A: Ultrasonography of the right thigh confirmed the diagnosis of pseudoaneurysm of LFCA (white arrow). B: The color Doppler image illustrates a typical characteristic of the yin-yang sign within the pseudoaneurysm sac.

An ultrasonography of the right thigh revealed a heterogenous collection at the anterolateral aspect, measuring 4.7 cm x 7.9 cm x 11.8 cm (AP x Wt x CC), consistent with a hematoma that communicates with the LFCA (Figure 3). Subsequent CT angiography confirmed the presence of a pseudoaneurysm arising from the descending branch of the LFCA of the profunda femoris, measuring 1.2 cm x 1.4 cm x 1.4 cm (AP x Wt x CC), accompanied by surrounding hematoma.

Following a 40-minute session of ultrasound-guided compression, the pseudoaneurysm reformed upon the release of compression. Following discussions with both the vascular surgeon and interventional radiologist, the patient proceeded with embolization of the LFCA pseudoaneurysm, performed by the interventional radiology team.

**Figure 4:**
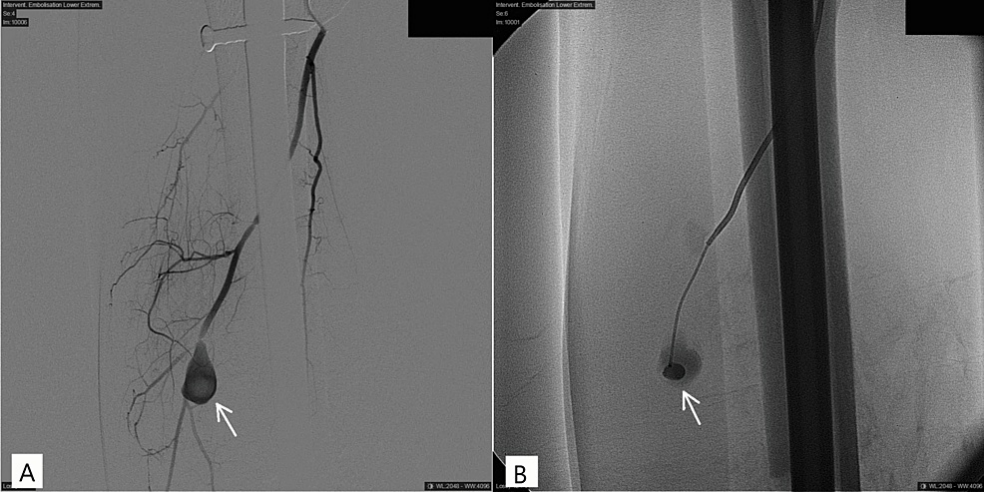
Pre- and post-angiography images (A and B) depict the guided embolization of a false aneurysm in a branch of the lateral femoral circumflex artery (LFCA) (white arrow).

A 5 Fr Cobra catheter was advanced through the right common femoral artery to reach the right LFCA. Subsequently, 1.5 ml of diluted Hystoacryl® glue was injected to secure the pseudoaneurysm (Figure 4). In this case, endovascular glue embolization was chosen as our interventional radiologist is trained in this procedure. Additionally, due to cost constraints, thrombin was not available at our center. Glue embolization was preferred over coil embolization due to the small size of the pseudoaneurysm.

The procedure was successful, as evidenced by the improvement in pain and the reduction in the size of the swelling. The patient was discharged in good condition after three days. Six weeks later, a follow-up CT angiography revealed the resolution of the false pseudoaneurysm of LFCA with no evidence of recurrence. Distal pulses in the right lower limb were both good in volume compared to the contralateral limb.

## Discussion

Femoral shaft fractures are common, especially in high-energy trauma, with an annual incidence reported at approximately 10 per 100,000 person-years [7]. Intramedullary nailing is a commonly performed and safe procedure for femoral shaft fractures, with entry points through either the greater trochanter or piriformis fossa. While complications related to bone, such as iatrogenic neck of femur fracture, avascular necrosis of the femoral head, and non-union, are known, vascular complications are rare but can occur in this procedure. This case report emphasizes the possibility of vascular injury, even when precautions are taken with great care.

Iatrogenic pseudoaneurysm may occur as a result of the sharp ends of guide wires, drill bits, or screws passing through an artery [8-10]. In this case, we postulate two possible mechanisms for the formation of the false aneurysm. First, it could be a result of the displacement of the fracture itself during the trauma. The initial plain radiographs indicated that the distal femur fragment was overlapping and displaced laterally, corresponding to the location of the pseudoaneurysm. While uncommon, late presentations of vascular injuries are not rare. Second, manual manipulation during surgery aimed at aiding reduction may cause the bone ends to injure the branch of the LFCA, and the traction force during reduction might contribute to tearing the vessel apart.

In cases where a pseudoaneurysm is suspected, the diagnosis can be confirmed through Doppler ultrasound or, for a more specific assessment, CT angiography.

Treatment options for pseudoaneurysms range from conservative management to endovascular intervention or open surgery. Small, asymptomatic lesions (less than 2 cm) may be observed, as spontaneous recovery might occur within four to six weeks [11]. However, active intervention is warranted for large and symptomatic lesions. With the advancements in current technology, open repair can often be avoided. Nevertheless, endovascular interventions are not without complications, including post-embolic syndrome, infection, and tissue necrosis. These risks can be minimized by experienced interventional radiologists using proper techniques and modern instruments, such as microcatheters and embolic agents.

## Conclusions

While intramedullary nailing is generally considered a safe procedure, suspicion of a pseudoaneurysm should arise if a patient presents with painful swelling post-surgery. An understanding of the anatomy, coupled with the characteristic history and examination findings of LFCA pseudoaneurysms, can guide clinicians in planning investigations and making a diagnosis following antegrade intramedullary device placement for midshaft femoral fractures. Early detection of this pathology is crucial to preventing further complications. Glue embolization of the artery should be considered as one of the treatment options, as it has shown favorable outcomes with reduced morbidity.
